# Severe Primary Raynaud's Disease Treated with Rituximab

**DOI:** 10.1155/2016/2053804

**Published:** 2016-08-29

**Authors:** Mohammed Shabrawishi, Abdurahman Albeity, Hani Almoallim

**Affiliations:** ^1^Department of Internal Medicine, King Faisal Specialist Hospital and Research Center, Jeddah, Saudi Arabia; ^2^Department of Internal Medicine, Medical College, Umm Alqura University, Makkah, Saudi Arabia

## Abstract

Raynaud's phenomenon refers to reversible spasms of the peripheral arterioles that can be primary Raynaud's phenomenon (PRP) or secondary Raynaud's phenomenon (SRP) to underlying connective tissue disease, both of which are characterized by a triphasic color response triggered by cold exposure or stress. PRP is typically a benign disease, whereas SRP may progress into digital ulcers and/or gangrene. Here, we report a case of a 55-year-old female diagnosed with PRP 7 years ago. Treatment with first-line agents, including calcium channel blocker, aspirin, and phosphodiesterase inhibitor, did not control her symptoms, which progressed to digital ulceration and gangrene. There were no symptoms of underlying autoimmune disease or malignancy, and autoimmune, serology, and immunology test results were normal; a biopsy of her left little finger was negative for vasculitis. Development to critical digital ischemia necessitated treatment with intravenous iloprost and heparin infusion followed by angioplasty, which led to a partial improvement. Due to persistent symptoms, rituximab therapy was initiated and two cycles induced a complete resolution of symptoms.

## 1. Introduction

Primary Raynaud's phenomenon (PRP) refers to symmetrical, vasospastic episodes of peripheral arterioles in the fingers and toes that are characterized by triphasic color changes exaggerated by physical, chemical, or emotional triggers and are typically associated with pain and/or numbness [1]. Raynaud's phenomenon is classified into primary Raynaud's phenomenon (PRP) and secondary Raynaud's phenomenon (SRP) according to the underlying etiology, such as various connective tissue diseases. PRP is typically a benign condition with an estimated prevalence of 4.85% among the general population [1]. However, transition to SRP is not uncommon and features, including late onset disease, abnormal nail fold capillaries, positive antinuclear antibody (ANA), or other autoantibodies and digital ischemia or ulceration, may predict it [2, 3]. The pathogenesis of RP is complex, and various vascular, neural, and intravascular mechanisms contribute to it [4]. The role of autoantibodies remains unclear; however, several studies have investigated this [5–9]. Nonpharmacologic lifestyle modifications and calcium channel blockers are the first-line treatment. Other medications, including phosphodiesterase type 5 inhibitors, endothelin antagonists, and prostaglandin derivatives, may be used in severe cases [10].

## 2. Case Report

The present patient, a 55-year-old Saudi female, was diagnosed with PRP in 2009. PRP initially manifested as classic bilateral discoloration of the fingers ranging between pallor, bluish, and reddish, with mild pain and numbness. The patient did not suffer from joint pain, swelling, or deformity and did not exhibit skin rash, oral ulcers, or dysphagia. Her symptoms were exacerbated by cold weather and stress. The patient is a lifelong nonsmoker. She was initially treated with aspirin, nifedipine, and prednisolone by another health facility, but no notable improvement was observed. Upon our initial assessment, she exhibited no features of connective tissue disease and the physical examination was remarkable for bilateral cyanosis of the fingertips, with a left middle finger ulcer, whereas a lower limb examination revealed bluish discoloration of the right and left second and third toe tips. Distal pulse and motor and sensory evaluations were normal. Laboratory investigation for autoimmune analysis and serology and malignancy screening were normal (Table 1).

The patient was admitted to our hospital in July 2010 with severe digital pain and ulceration. Magnetic resonance angiogram of her upper extremities did not show any features of vasculitis, aneurysm, or stenosis. Computed tomography (CT) scanning of the aortic arch demonstrated a normal aorta and normal branches with no obvious vascular abnormality. CT angiogram demonstrated attenuated, irregular right and left ulnar arteries. The patient underwent bilateral selective ulnar angiography and distal angioplasty, which indicated distal disease at the level of the palmar arches. Biopsy of the left little finger showed no significant histopathological abnormalities and was negative for vasculitis.

**Figure 1:**
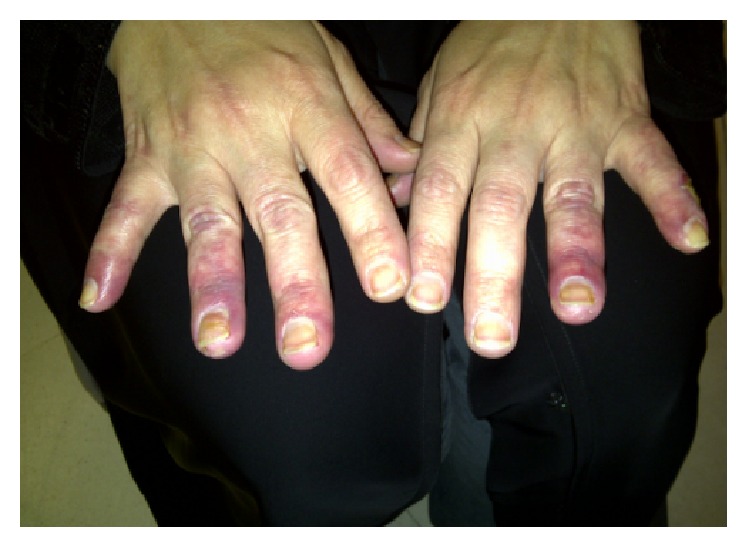
Prior to rituximab therapy.

Prednisolone therapy was discontinued, and the patient was given a trial of sildenafil (12.5 mg) twice a day, as bosentan was intolerable due to side effects (nausea and dizziness). Disease progression was noted and the patient suffered from severe digital pain and bilateral ulceration of the tips of her fingers. These symptoms required another admission to the hospital and treatment with intravenous (IV) heparin infusion and IV iloprost for a total of 7 days, which led to a minimal improvement in her symptoms. Due to persistent symptoms (Figure 1), rituximab therapy was initiated and she received first cycle in 2012 (1 g two weeks apart). Follow-up after 2 months indicated a significant improvement in her signs and symptoms.

**Figure 2:**
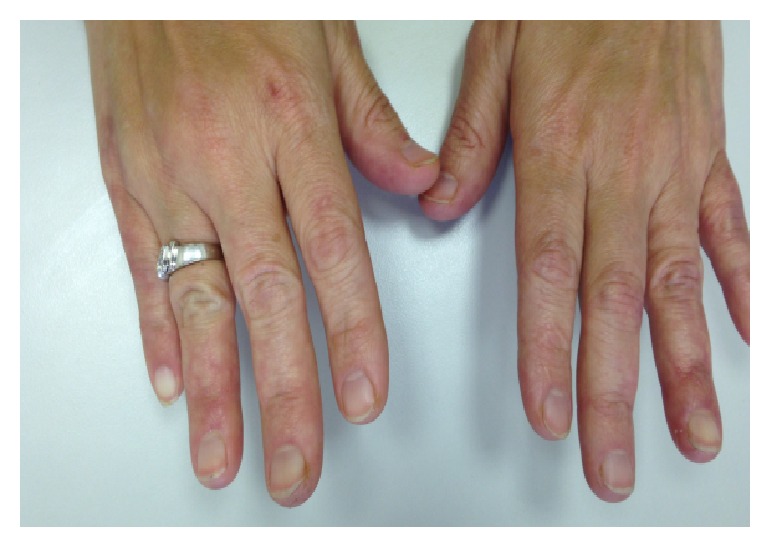
Following the first cycle of rituximab.

**Figure 3:**
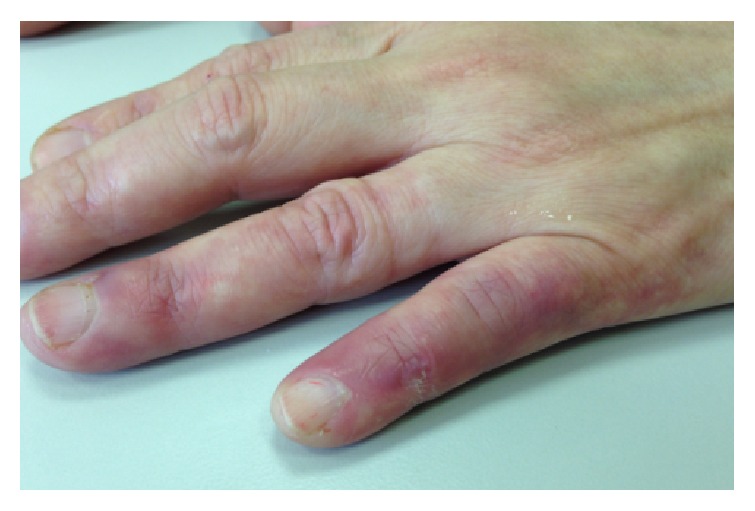
Recurrence of symptoms.

**Figure 4:**
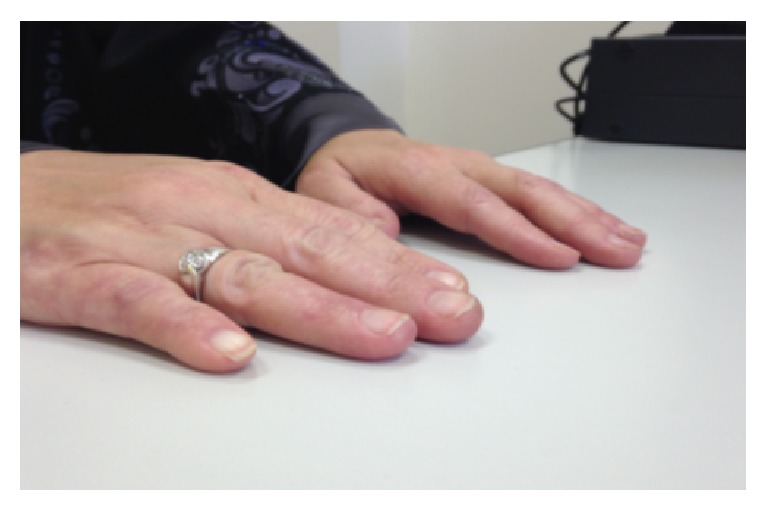
Following the second cycle of rituximab therapy.

Follow-up after six months of rituximab therapy indicated a complete resolution of the digital ischemia (Figure 2). In January 2015, the patient experienced a recurrence of her previous symptoms (Figure 3), which were resolved by a second cycle of rituximab (375 mg/m^2^ weekly for 4 weeks). As of the most recent follow-up in April 2016, the patient remains in full remission (Figure 4).

## 3. Discussion

The present case illustrated the successful use of rituximab in a patient with severe PRP that was resistant to alternative therapeutic management options. The initial and follow-up investigations did not define a secondary cause; however, as our patient exhibited more than one feature that may favor the transition to SRP, including age >40, digital ischemia, and ulceration [2, 3], long term follow-up is required. Although it remains unclear whether autoantibodies have a major role in the pathogenesis of primary or secondary RP, two previous studies have established the presence of antibodies against type IV collagen in patients with PRP [5, 6], which may explain the response to rituximab in the present case. Rituximab was associated with satisfactory outcomes in patients with refractory antibody-mediated antiglomerular basement membrane disease [11], which suggests targeting of the antigen alpha-3 chain of type IV collagen [12, 13]. Furthermore, two case reports evaluated the use of rituximab in SRP associated with connective tissue diseases. Resolution of symptoms and reduction of ANA titer were described in one patient [14], whereas the other patient's PRP symptoms did not improve despite depletion of B cells at two- and six-month intervals [15]. Further studies are required to conclude the role and efficacy of this novel treatment in PRP and secondary RP.

## Figures and Tables

**Table 1:** 

Test	Patient results by year			Normal range
	2010	2012	2015	
ESR (mm/Hr)	35	27	30	<30
CRP (mg/L)	1.88	1.92	1.73	0–5
ANA	Negative		Negative	<1 : 20
Anti-DsDNA (U/mL)	18.2			0–20
Anti-Smith antibody (U/mL)	<3.1			0–5
Anti-SSA (Ro) antibody (U/mL)	<3.1			0–4
Anti-SSB (La) antibody (U/mL)	<3.1			0–4
Anti-SCL-70 antibody (U/mL)	<3			0–3
C-ANCA (U/mL)	<3.1	<3.1		0–10
P-ANCA (U/mL)	3.5	4.8		0–6
Anti-phosphatidylserine IgM and IgG (GPL/mL)	Negative	Negative		0–10
Anti-B2-glycoprotein IgM and IgG (GPL/mL)	Negative	Negative		0–12
Anti-cardiolipin IgM and IgG (GPL/mL)	Negative	Negative		<10
Cyclic citrullinated peptide antibody (U/mL)	<0.5		<1.0	0–4.9
Rheumatoid factor (IU/mL)	7.4		<10	0–14
Cryoglobulin	Absent		Absent	
Serum protein electrophoresis	Normal pattern		Normal pattern	
Urine protein electrophoresis	Normal pattern		Normal pattern	

ESR: erythrocyte sedimentation rate; CRP: C-reactive protein; ANA: antinuclear antibody; SSA: Sjögren's-syndrome-related antigen A; SSB: Sjögren's-syndrome-related antigen B; SCL: scleroderma; C-ANCA: cytoplasmic antineutrophil cytoplasmic antibodies; P-ANCA: perinuclear antineutrophil cytoplasmic antibodies.
